# Viscoelastic properties of human and bovine articular cartilage: a comparison of frequency-dependent trends

**DOI:** 10.1186/s12891-016-1279-1

**Published:** 2016-10-06

**Authors:** Duncan K. Temple, Anna A. Cederlund, Bernard M. Lawless, Richard M. Aspden, Daniel M. Espino

**Affiliations:** 1 Department of Mechanical Engineering, University of Birmingham, Birmingham, B15 2TT UK; 2 Institute of Medical Sciences, Foresterhill, University of Aberdeen, Aberdeen, UK

**Keywords:** Articular cartilage, Bovine, Frequency, Human, Loss, Modulus, Storage, Viscoelastic properties

## Abstract

**Background:**

The purpose of this study was to compare the frequency-dependent viscoelastic properties of human and bovine cartilage.

**Methods:**

Full-depth cartilage specimens were extracted from bovine and human femoral heads. Using dynamic mechanical analysis, the viscoelastic properties of eight bovine and six human specimens were measured over the frequency range 1 Hz to 88 Hz. Significant differences between bovine and human cartilage viscoelastic properties were assessed using a Mann–Whitney test (*p* < 0.05).

**Results:**

Throughout the range of frequencies tested and for both species, the storage modulus was greater than the loss modulus and both were frequency-dependent. The storage and loss moduli of all human and bovine cartilage specimens presented a logarithmic relationship with respect to frequency. The mean human storage modulus ranged from 31.9 MPa to 43.3 MPa, while the mean bovine storage modulus ranged from 54.0 MPa to 80.5 MPa; bovine storage moduli were 1.7 to 1.9 times greater than the human modulus. Similarly, the loss modulus of bovine cartilage was 2.0 to 2.1 times greater than human. The mean human loss modulus ranged from 5.3 MPa to 8.5 MPa while bovine moduli ranged from 10.6 MPa to 18.1 MPa.

**Conclusion:**

Frequency-dependent viscoelastic trends of bovine articular cartilage were consistent with those of human articular cartilage; this includes a similar frequency dependency and high-frequency plateau. Bovine cartilage was, however, ‘stiffer’ than human by a factor of approximately 2. With these provisos, bovine articular cartilage may be a suitable dynamic model for human articular cartilage.

## Background

In a healthy joint, articular cartilage provides a smooth load-bearing surface. In the lower limbs it sustains physiological stresses estimated to be in the range of 1–6 MPa during level walking [1]. However, these stresses increase with standard daily activities [2], or activities such as squatting [3], up to and beyond 10 MPa.

It has been hypothesized that a sub-group of the population with rapid heel-strike rise times may be predisposed to developing osteoarthritis [4] although evidence supporting this is proving elusive. This sub-group had heel-strike rise times of around 5–25 ms [5], compared with rise-times of 100–150 ms for the general population [6]. These rise-times have been calculated as being equivalent to frequencies of 3–5 Hz for the latter case, but of up to 90 Hz for rapid heel-strikes [7]. It has recently been shown that increasing the loading frequency alone, independent of load, predisposes cartilage to damage [8]. This was consistent with studies predicting increased failure with frequency, inferred from the measured frequency-dependency of viscoelastic properties [7].

Dynamic Mechanical Analysis (DMA) is a method which enables the characterisation of viscoelastic properties under dynamic loading and can be combined with a frequency sweep. A viscoelastic material can be characterised in terms of its storage and loss moduli. The storage modulus, *E’*, characterises the material’s ability to store energy for elastic recoil; while, the loss modulus, *E”*, characterises the material’s ability to dissipate energy. *E’* and *E”* are related to the dynamic (complex) modulus, E*, and phase angle, *δ*, of a viscoelastic material according to Eqs. 1 and 2 [9].

1 $$ \left|{E}^{*}\right|=\sqrt{E{\mathit{\hbox{'}}}^2+E{"}^2} $$

2 $$ \delta =ta{n}^{\mathit{\hbox{-}} 1}\left(\frac{E"}{E\mathit{\hbox{'}}}\right) $$

Several recent studies have used DMA to assess frequency-dependency of viscoelastic properties of bovine articular cartilage. For example, the storage modulus was found to plateau at frequencies above 20 Hz. This was attributed to a glassy transition at around 10 Hz [7]. The storage modulus is generally greater than the loss modulus at around gait-relevant frequencies and above, albeit tending towards parity below these frequencies [10]. Further, storage and loss moduli are susceptible to changes in cartilage hydration [11] and vary with thickness [12]. Freezing, however, does not alter its viscoelastic properties [13].

Bovine cartilage is commonly chosen as an experimental model [7, 10, 12, 14–18]. Studies assessing its suitability as a model for human cartilage have mostly focused on elastic properties based on creep tests [19], joint geometry and cartilage thickness [15] or ultrastructure [18]. Others have questioned this due to differences in zonal variation of structure and response to impact loading [20], cellularity and biological response to mechanical stimuli [21]. Therefore, it is currently unknown whether frequency-dependent trends of viscoelastic properties of bovine cartilage are comparable with those of human articular cartilage.

The aim of this study was to assess the benefits and limitations of using bovine articular cartilage as a dynamic model for human articular cartilage. Viscoelastic properties of human and bovine cartilage have been characterised over a range of physiological and pathophysiological loading frequencies.

## Methods

### Human specimens

Three human femoral heads (3 female, ages 69, 78 and 83) were retrieved from surgery following traumatic fracture of the femoral neck. Ethical approval was given by the North of Scotland Research Ethics Committee though the Grampian Biorepository and written consent for the use of their tissue for research was obtained from the patients. The femoral heads were wrapped in moist gauze and stored at – 20 °C.

On the day prior to testing the femoral heads were thawed and the gauze removed. India ink (Loxley Art Materials, Sheffield, UK) was applied to, and rinsed off, the articular cartilage to identify surface lesions [22, 23] and regions showing pre-existing lesions were not selected for testing. Six cylindrical cores (5 mm in diameter) of femoral human articular cartilage were cored using a cork borer, two from each femoral head. They were gently subtracted from the bone using a sharp scalpel. The heights of the samples varied. The samples were then stored in physiological strength phosphate-buffered saline (Sigma-Aldrich, Dorset, UK) until testing took place.

### Bovine specimens

Three bovine femurs, from healthy cows aged 18–30 months, were obtained from a supplier (Dissect Supplies, Birmingham, UK). Upon arrival at the laboratory, the femoral heads were carefully dissected from the femur. The femoral heads were individually wrapped in tissue paper soaked in Ringer’s solution and heat-sealed in plastic bags. The femoral heads were then stored at – 40 °C until the day of testing.

On the day of testing, the femoral heads were removed from the plastic bags and wrapped in tissue saturated in Ringer’s solution. Similarly to the human specimens, India ink (Loxley Art Materials, Sheffield, UK) was applied to, and rinsed off, the articular cartilage to identify surface lesions [22, 23]. Regions showing pre-existing lesions were not selected for testing. Large scale damage was not evident which suggests that the joints were not osteoarthritic. Both human and bovine cartilage specimens used were considered healthy and skeletally mature. Thus, cartilage specimens from both human and bovine joints were randomly selected in terms of location on the joint, with the criteria that the articulating cartilage surface be intact. Particularly for human joints, though, the best preserved cartilage tended to be primarily from the superior region. It is noted that the cartilage surface can be used as a guide to the degradation of the underlying cartilage [24].

Eight cylindrical-shaped specimens of bovine articular cartilage, from the three femoral heads, were obtained. Bovine cartilage was readily available leading to more samples being tested than for human cartilage. Therefore, three tissue specimens were taken from two bovine femoral heads while only two were taken from a third femoral head. All specimens were obtained using a cork borer with a medical scalpel used to isolate the cartilage from the subchondral bone [25–27]. All cylindrical specimens were 5 mm in diameter, but varied in thickness. The specimens were thawed to room temperature, saturated with Ringer’s solution; this ensured full hydration before testing. Once defrosted, cartilage samples were placed on an aluminium base, ready for DMA. Freezing and thawing has been shown not to alter the dynamic properties of cartilage [13].

### DMA frequency sweep

The viscoelastic properties of human and bovine cartilage were measured using a Bose ElectroForce 3200 testing machine and Bose WinTest 4.1 DMA software (Bose Corporation, Electroforce Systems Group, Minnesota, USA). Similar to previous cartilage studies [7, 10–12], asinusoidal, compressive force, between 16 N and 36 N, was applied to each specimen under unconfined conditions. Specimens were loaded using a 20 mm diameter compression plate, i.e. the loading device was much larger than the cartilage samples tested [28], which came into contact with the articulating surface of samples. Since the specimens were 5 mm in diameter, the peak stress induced was 1.8 MPa, similar to the estimated cartilage physiological peak stress during walking of 1.7 MPa [1]. All specimens were tested in air at room temperature. The testing procedure only requires a few minutes and dehydration over such a short time-scale is not anticipated, particularly given that only the rim of the cylindrical core is exposed to air.

To achieve a dynamic “steady-state”, which for *ex vivo* cartilage occurs after around 1200 to 4500 preconditioning cycles [7, 29, 30], two preload conditions, 25 Hz and 50 Hz, were applied before the frequency sweep. These precondition cycles were applied for 60 s each with a 60 s rest period between cycles [7, 11, 12]. After preconditioning, eight different sinusoidal frequencies (1, 8, 10, 12, 29, 49, 71, and 88 Hz) were applied to the cartilage specimens; this range has been described in previous studies [7]. For each frequency, the WinTest DMA software performed Fourier analyses of the sinusoidal force and displacement waves. From the Fourier transforms, the magnitudes of the load (F*) and displacement (d*), the phase lag (*δ*) and the frequency are calculated. The viscoelastic properties, complex stiffness (k*), storage stiffness (*k’*) and loss stiffness (*k”*) were then calculated using [31]:

3 $$ {k}^{*}=\frac{F^{*}}{d^{*}} $$

The storage (*E’*) and loss (*E”*) moduli were then calculated according to Eqs. 4 and 5, using a shape factor, *S,* Eq. 6 [7].

4 $$ E\mathit{\hbox{'}}=\frac{k^{*} cos\delta}{s} $$

5 $$ E"=\frac{k^{*} sin\delta}{s} $$

6 $$ S=\frac{\pi {d}^2}{4h} $$

Here, *d* is the sample diameter (5 mm), and *h* is the specimen thickness which was measured for human and bovine samples. An established needle technique, previously described [6, 7, 12, 13, 15], was used to measure the thickness of bovine cartilage. Briefly, a sharp needle is pushed through the cartilage surface and the thickness is measured using the testing machine’s displacement transducer (1 μm resolution). The needle technique, however, causes damage to the cartilage sample. The human cartilage samples were required for another study, therefore, a Vernier Calliper was used to measure the thickness of the human specimens (0.1 mm resolution).

### Statistical analysis

Statistical analyses were performed by using SigmaPlot 12.0 (SYSTAT, San Jose, CA, USA). For bovine cartilage, *E’* has been shown to follow a logarithmic trend [7, 12] of the form:

7 $$ E\mathit{\hbox{'}}=A\; 1n(f)+B $$

where *A* defines the gradient of *E’* against the natural logarithm of *f*, the frequency in Hz, and *B* is the intercept.

Previously, *E”* for bovine cartilage on-bone has been found to be frequency-independent [7]. However, for this study cartilage was tested off-bone. Therefore, regression analysis was performed to evaluate the best trend, if any, for *E”*. The statistical significance of the curve fit was assessed for both *E’* and *E”*. The storage modulus and loss modulus between bovine and human cartilage, for each frequency tested, were compared using a Mann–Whitney test. Statistical results with *p* < 0.05 were considered significant. Power analyses, of the Mann–Whitney tests, were performed with G*Power 3.1 (Heinrich-Heine-University, Dusseldorf, Germany) [32] to evaluate the chances of a Type II error being present. Power analysis results greater than 0.80 were deemed sufficient.

## Results

**Fig. 1:**
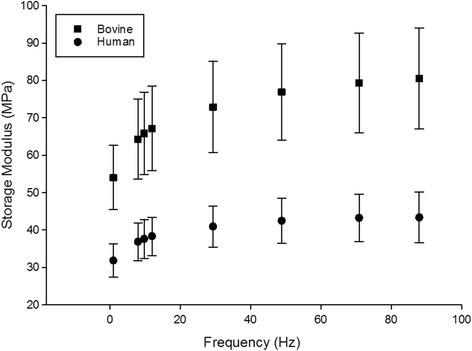
The storage modulus (*E’*) of human and bovine cartilage (mean ± SD)

**Fig. 2:**
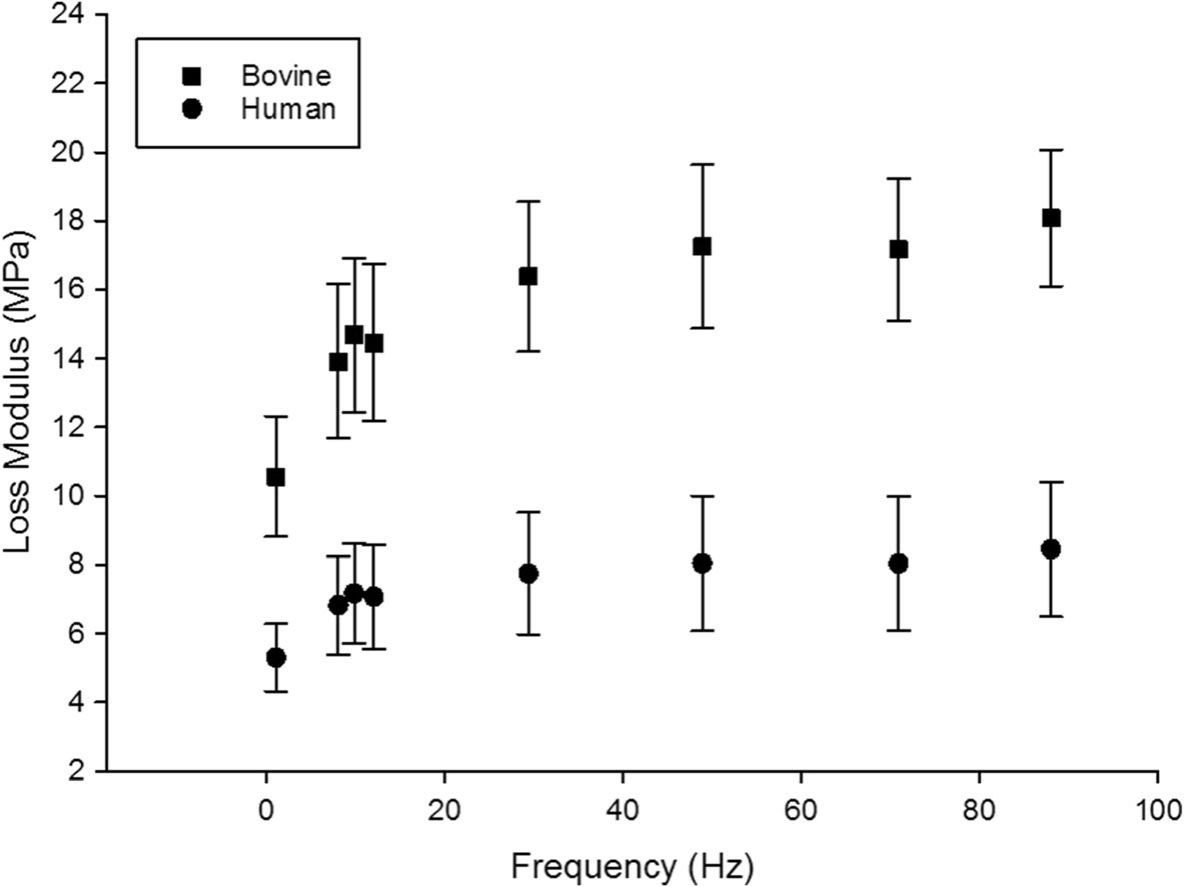
The loss modulus (*E”*) of human and bovine cartilage (mean ± SD)

For all frequencies tested, *E’* of human and bovine cartilage was greater than *E”* (Figs. 1 and 2). The frequency-dependency of *E’* for both human and bovine cartilage is shown in Fig. 1. The bovine cartilage mean storage modulus ranged from 54.0 MPa at 1 Hz to 80.5 MPa at 88 Hz. This range was approximately 1.7-1.9 times greater than the human cartilage mean storage modulus (31.9 MPa to 43.3 MPa). Bovine storage and loss moduli were statistically different (*p* < 0.05), for every tested frequency, when compared to human storage and loss moduli, respectively.

For all samples tested, *E’* followed a logarithmic (Eq. 7) trend with frequency (*p* < 0.05, *r* ^*2*^ ≥ 0.97; Table 1). The two constants, *A* and *B,* used to describe the frequency-dependency were significantly greater for bovine than for human cartilage (Table 1). This trend included a lowest value for *E’* at 1 Hz, and tending to reach a plateau above 20 Hz (Fig. 1).

**Table 1:** Frequency-dependency of *E’* (Eq. 7) for individual specimens

Specimen	Thickness (mm)	*A (MPa)*	*B (MPa)*	*r* ^*2*^	*p* value
Bovine 1	1.20	4.58	59.26	0.99	0.0001
Bovine 2	1.18	6.77	54.93	0.99	0.0001
Bovine 3	1.15	8.07	57.54	0.99	0.0001
Bovine 4	0.84	5.40	53.43	0.99	0.0001
Bovine 5	0.88	3.52	34.49	0.98	0.0001
Bovine 6	1.28	6.46	51.47	0.98	0.0001
Bovine 7	1.18	6.71	63.09	1.00	0.0001
Bovine 8	1.15	7.19	47.69	0.98	0.0001
Bovine Mean	1.11 ± 0.16	6.09 ± 1.49	52.74 ± 8.77	0.99	0.0001
Human 1	1.0	3.4	39.6	0.99	0.0001
Human 2	1.3	2.9	29.3	1.00	0.0001
Human 3	1.8	2.2	29.8	0.99	0.0001
Human 4	2.0	2.6	33.1	0.99	0.0001
Human 5	1.1	3.2	29.8	0.99	0.0001
Human 6	1.7	1.8	28.3	0.97	0.0001
Human Mean	1.4 ± 0.4	2.7 ± 0.6	32.7 ± 4.2	1.00	0.0001

Constants *A* and *B* define trends for *E’* with frequency

*E”* was also found to be frequency-dependent for both bovine and human samples. This can be described using a relationship, similar to that for *E’*, with the logarithm of frequency (Eq. 8; *p* < 0.05, *r* ^*2*^ ≥ 0.90; Table 2). *E”* increased with frequency, with the lowest value at 1 Hz, 5.3 MPa for human and 10.6 MPa for bovine cartilage, rising to 8.5 MPa for human and 18.1 MPa for bovine tissue at 88 Hz. Bovine values were approximately twice those for human samples.

**Table 2:** Frequency-dependency of *E”* (Eq. 8) for individual specimens

Specimen	*C (MPa)*	*D (MPa)*	*r* ^*2*^	*p* value
Bovine 1	1.55	10.02	0.98	0.0001
Bovine 2	1.65	11.79	0.99	0.0001
Bovine 3	1.96	12.73	0.98	0.0001
Bovine 4	1.25	10.55	0.97	0.0001
Bovine 5	1.73	6.33	0.95	0.0001
Bovine 6	2.08	9.92	0.98	0.0001
Bovine 7	1.21	12.77	0.93	0.0001
Bovine 8	1.76	10.73	0.98	0.0001
Bovine Mean	1.65 ± 0.31	10.60 ± 2.06	0.99	0.0001
Human 1	1.0	6.3	0.99	0.0001
Human 2	0.4	6.2	0.91	0.0001
Human 3	0.8	3.9	0.99	0.0001
Human 4	0.5	6.0	0.92	0.0001
Human 5	1.0	5.9	0.99	0.0001
Human 6	0.2	4.3	0.90	0.0001
Human Mean	0.7 ± 0.3	5.4 ± 1.1	0.98	0.0001

Constants *C* and *D* define trends for *E”* with frequency

8 $$ E"=C\; 1n(f)+D $$

Here *C* defines the gradient of *E”* plotted against the natural logarithm of *f,* and *D* is the intercept. *C* and *D* were significantly greater for bovine cartilage, by factors of 2.5 and 2.0, respectively (Table 2).

## Discussion

This study compares the frequency-dependent viscoelastic properties of isolated human and bovine articular cartilage. The storage and loss moduli of both human and bovine articular cartilage follow a trend that is logarithmic with frequency. The moduli increase at low frequencies followed by a plateau above about 20 Hz; consistent with previous studies [7]. The frequency-dependent storage and loss moduli of bovine cartilage appear to follow a similar trend as those for human cartilage, but greater by a multiple of around 2. The ratio of storage to loss modulus is greater than that predicted from impact loading studies of around one [25]. However, a recent study has shown that as loading frequencies used for DMA go below 1 Hz the ratio of storage to loss modulus does tend towards parity [10].

The range and frequency-dependent trends for storage moduli obtained for bovine samples (in the range of 50 to 80 MPa) were comparable to previous ranges over a similar frequency-sweep. For example, storage moduli of bovine tibial plateau cartilage range from around 35 to 60 MPa [7] or 27 to 52 MPa [12]. Thus, storage moduli reported are consistent with previous studies, but critically the storage moduli of bovine and human cartilage follow the same frequency-dependent trend.

The increase in storage modulus as frequency increases from 1 Hz up to 90 Hz, seen for both human and bovine cartilage in this study, has been suggested as potentially predisposing cartilage to failure at higher frequencies. This was due to the increase in energy stored, as compared to dissipated, during cartilage deformation at higher frequencies, e.g. 20 – 90 Hz [7]. The frequency-dependent failure predicted by Fulcher at al. [7] is consistent with a recent study finding that the frequency of loading, alone, alters the extent of surface damage on bovine cartilage [8]. Further, the mechanical behaviour of bovine cartilage measured below 1 Hz is not representative of cartilage under loading conditions associated with gait [10]. Thus, from the consistency in trends of viscoelastic properties between human and bovine cartilage, from our current study, we would expect this trend to hold for human cartilage; however, the patterns of failure may well differ [20].

The loss modulus measured in this study demonstrated a frequency-dependency. This has not been found in recent studies using DMA [7, 10–12]. This may be a result of different test procedures used. In this present study, articular cartilage was tested after removal from its underlying bone, unlike previous studies which have included the subchondral bone. However, hysteresis can decrease when cartilage is on-bone, but increase when off-bone, with increased loading velocity [25]; such differences being due to the restraining effect of bone [33]. The hypothesis that the loss modulus remains frequency-independent when cartilage is on-bone but frequency-dependent when off-bone requires experimental verification. Importantly, though, loss moduli of bovine and human cartilage followed similar trends with frequency in this study.

In this present study, bovine cartilage has been found to be approximately twice as ‘stiff’ as human cartilage, in terms of both storage and loss moduli. This is in contrast to a study which has found human cartilage to be stiffer than bovine cartilage [15]. Taylor et al. [15] found the equilibrium modulus of human femoral head cartilage to be 4.89 MPa, as compared with 1.86 MPa for bovine cartilage. However, our findings are closer to another study [19]. Athanasiou et al. [19] found the aggregate moduli of human lateral (0.70 MPa) and medial (0.59 MPa) condylar cartilage to be lower than those of bovine cartilage (0.89 and 0.90 MPa, respectively). More detailed comparisons are not meaningful because both of these previous studies used creep tests, as opposed to the dynamic procedure used in this study. For example, creep tests have led calculations of a frequency-dependent loss, but not storage, modulus [34]. Comparisons are further hindered because the applied stresses in previous studies have been well below 1 MPa [15, 19]; to which the cartilage compressive modulus, which has a bimodal relationship with strain [35], is likely to be sensitive. Human cartilage being thicker than bovine cartilage is, though, consistent with storage and loss moduli decreasing with increased thickness of cartilage [12].

Clearly, there are limitations in using bovine cartilage as an experimental model for human cartilage. For example, Jeffrey and Aspden [20] found that bovine cartilage did not suffer the same pattern of damage as human cartilage when subjected to an impact load, possibly because it had a different collagen organisation. They also found that nominal stresses induced following impact loading were 1.9 times greater in bovine as compared to human cartilage. Furthermore, calculations of a maximum dynamic modulus, which was defined as the maximum value of the differentiated stress–strain curve, found bovine cartilage measurements (170 MPa) to be greater than those of human cartilage (85 MPa) by a multiple of 2 [20]. These differences in moduli are consistent with the findings in our present study, where storage and loss moduli of bovine cartilage are approximately twice those of human cartilage. However, in this present study, we have found that the 2 times multiple appears to remain constant throughout a range of frequencies relevant to physiological, and potentially patho-physiological, loading. Thus, the benefits of using a bovine model are that it follows the same frequency-dependency as human cartilage when loaded at relevant frequencies and stresses; whereas, the limitation is that measured moduli may be around twice those of human samples.

When evaluating the viscoelastic properties of bovine femoral head cartilage to human femoral head cartilage, a scaling factor of 2 appears appropriate. However, the caveat in the comparison is important for quantitative work, because the difference was statistically significant. Further, it may reflect differences in collagen organisation which may limit the extrapolation from bovine to human models of say failure patterns, already seen during single impact tests [20]. It should be noted, though, that human samples were from older donors. The use of older human tissues may not be ideal because of possible age related changes. However, this is a common limitation when working with human tissues as acquiring healthy specimens, from the younger population, is difficult [36]. In fact, the availability of healthy human specimens is limited. For this study, for example, three human femoral heads were retrieved. Although testing the same number of bovine and human tissue samples was not deemed necessary, the number of independent observations were maintained constant [37]. Hence, bovine samples were also obtained from three femoral heads.

A limitation of this study is that different techniques were used to measure the thickness of human and bovine cartilage. Thus, it is possible that there is a measurement bias between human and bovine cartilage thickness. This bias would alter the shape factor used to calculate moduli (Eqs. 4 and 5), but not the frequency-dependent trends. However, large errors are not anticipated because the lowest measurement resolution was that from the use of a Vernier calliper, for human samples, with a resolution of 0.1 mm. The needle technique used to measure bovine cartilage thickness has greater precision as it is measured with the testing machine’s displacement transducer, with resolution of 1 μm. The thickness of human samples was not measured using the needle technique, because it risks damaging the specimen and the samples were required for further tests. Regardless, our measurements of human cartilage thickness of approximately 1.4 mm are consistent with human femoral head cartilage thickness ranging from 1.35 to 2.0 mm [38] and human femoral head cartilage being thicker than bovine cartilage [15, 20]. Given this difference in cartilage, samples were not matched for thickness during our testing. Such a selection process would be misleading as it would introduce bias into the comparison, and might not have provided a measure of the intrinsic differences in material properties of cartilage on human and bovine femoral heads.

Along with differences in cartilage thickness, bovine cartilage has some known differences in ultrastructure [18, 20]. These differences include the arrangement of collagen around the transition zone. In terms of macro-scale DMA, though, such differences were not reflected in frequency-dependent trends. However, such differences may be of greater relevance to ultra- and nano-scale deformation which may be of relevance to lubrication mechanics [39]. In particular, nano-DMA tests [40–42] may be more sensitive to the collagen arrangement around the transition and surface layers.

## Conclusions

Frequency-dependent viscoelastic trends of bovine articular cartilage are similar to those of human articular cartilage, including a similar frequency dependency and high-frequency plateau. Bovine cartilage, however, was ‘stiffer’ than human cartilage by a factor of approximately 2. With these provisos, bovine articular cartilage may be a suitable dynamic model for human articular cartilage.

## Abbreviations

*d**: Magnitude of the displacement obtained from Fourier transform
*d*: Sample diameter
*DMA*: Dynamic mechanical analysis
*E**: Complex modulus
*E'”*: Loss modulus
*E’*: Storage modulus
*F**: Magnitudes of the load obtained from Fourier transform
*f*: Frequency
*h*: Specimen thickness
*k**: Complex stiffness
*k’*: Storage stiffness
*k”*: Loss stiffness
*S*: Shape factor
*δ*: Phase angle
